# Multiple *M. tuberculosis* Phenotypes in Mouse and Guinea Pig Lung Tissue Revealed by a Dual-Staining Approach

**DOI:** 10.1371/journal.pone.0011108

**Published:** 2010-06-14

**Authors:** Gavin J. Ryan, Donald R. Hoff, Emily R. Driver, Martin I. Voskuil, Mercedes Gonzalez-Juarrero, Randall J. Basaraba, Dean C. Crick, John S. Spencer, Anne J. Lenaerts

**Affiliations:** 1 Department of Microbiology, Immunology and Pathology, Colorado State University, Fort Collins, Colorado, United States of America; 2 School of Medicine, University of Colorado Denver, Denver, Colorado, United States of America; CNRS/Université de Toulouse, France

## Abstract

A unique hallmark of tuberculosis is the granulomatous lesions formed in the lung. Granulomas can be heterogeneous in nature and can develop a necrotic, hypoxic core which is surrounded by an acellular, fibrotic rim. Studying bacilli in this *in vivo* microenvironment is problematic as *Mycobacterium tuberculosis* can change its phenotype and also become acid-fast negative. Under *in vitro* models of differing environments, *M. tuberculosis* alters its metabolism, transcriptional profile and rate of replication. In this study, we investigated whether these phenotypic adaptations of *M. tuberculosis* are unique for certain environmental conditions and if they could therefore be used as differential markers. Bacilli were studied using fluorescent acid-fast auramine-rhodamine targeting the mycolic acid containing cell wall, and immunofluorescence targeting bacterial proteins using an anti-*M. tuberculosis* whole cell lysate polyclonal antibody. These techniques were combined and simultaneously applied to *M. tuberculosis in vitro* culture samples and to lung sections of *M. tuberculosis* infected mice and guinea pigs. Two phenotypically different subpopulations of *M. tuberculosis* were found in stationary culture whilst three subpopulations were found in hypoxic culture and in lung sections. Bacilli were either exclusively acid-fast positive, exclusively immunofluorescent positive or acid-fast and immunofluorescent positive. These results suggest that *M. tuberculosis* exists as multiple populations in most conditions, even within seemingly a single microenvironment. This is relevant information for approaches that study bacillary characteristics in pooled samples (using lipidomics and proteomics) as well as in *M. tuberculosis* drug development.

## Introduction

*Mycobacterium tuberculosis*, the causative agent of the disease tuberculosis (TB), has proved extremely difficult to treat for more than a century. Almost 1.8 million people died from TB in 2007 [1] despite vast campaigns undertaken by national and international agencies to control and eliminate this infection. The success of this bacillus in causing TB partly resides in its ability to adapt to the various microenvironments within the human host which renders the bacilli refractory to drug treatment [2], [3].

When *M. tuberculosis* first enters the lung, the bacilli are phagocytosed by alveolar macrophages and infection is then contained by host cellular recruitment into the formation of granulomas [4]. In nonhuman primates, rabbits and guinea pigs a heterogeneity of lesions is observed comprising of initial hypoxic, necrotic primary granulomas and then secondary inflammatory lesions which originate at a later stage after dissemination [5], [6], [7], [8]. This hypoxic environment is one of many which *M. tuberculosis* adapts to in order to survive. When grown under hypoxic conditions *in vitro* in the laboratory, *M. tuberculosis* alters its replication rate [9] and also changes its metabolism [10], [11], [12], [13]. The adaptation to hypoxic conditions, and to nutrient starvation [14], is thought to be, at least partly, responsible for its ability to survive in a latent state for long periods in humans [9] [10], [15], [16].

The mechanisms by which the bacilli survive in the granuloma are poorly understood. To date, there are only few studies published which investigate *M. tuberculosis* within granulomas which is most likely due to technological difficulties of extracting *M. tuberculosis* from tissue and performing proteomic and lipidomic studies on limited bacterial numbers. The goal of this study was to investigate *M. tuberculosis* in their *in vivo* environment by using differential staining techniques that target specific components of the *M. tuberculosis* bacillus, and whether certain bacillary populations could be identified in the different microenvironments tested *in vitro* and *in vivo*. Current published methods used to visualize and locate bacteria within infected tissue allow for detection of: 1) bacterial lipid by acid-fast staining [Ziehl-Neelsen (ZN) and auramine-rhodamine (AR) [17]], 2) bacterial surface proteins by immunohistochemistry (IHC) or immunofluorescence (IF) [18], [19], [20] and 3) bacterial nucleic acid by *in situ* hybridization (ISH) [21], [22]. IHC and IF both use antibodies directed against a desired target but the methods mainly differ in the use of a secondary detection step as IHC uses a chromogen for color visualization whilst IF uses fluorophores.

Both IHC and acid-fast staining visualize specific targets, therefore we hypothesized that only certain subpopulations of *M. tuberculosis* would be detected by each individual detection technique as numerous published studies have shown that the cell wall of *M. tuberculosis* can undergo alterations under certain *in vitro* and *in vivo* conditions. Deb *et al* showed that *M. tuberculosis* can lose its acid-fastness under multiple stresses *in vitro* [23]. Another *in vitro* study showed that altered mycolic acid ratios along with a shortened mycolic acid chain length render *M. tuberculosis* acid-fast negative [24]. In macrophages, levels of certain mycolic acids of *M. tuberculosis* are substantially altered [25]. In mice, it has also been demonstrated that *M. tuberculosis* can lose its ability to retain the Ziehl Nielsen acid-fast stain [19], [20]. Furthermore, the cell wall of *M. tuberculosis* has been described to thicken when grown under hypoxic conditions *in vitro* as was shown by transmission electron microscopy [26]. In addition to cell wall alterations, it has been shown that *M. tuberculosis* changes its transcription profile significantly under different environmental conditions. Low oxygen tension, NO, or CO induce the transcription of a set of genes known as the DosR regulon, which allow the bacteria to survive under these conditions [27], [28], [29]. Under anaerobic conditions, RNA and protein synthesis are significantly reduced and the bacilli enter a dormant state [10], [13], [30]. In macrophages, *M. tuberculosis* upregulates an iron-scavenging pathway and induces anaerobic respiration along with the dormancy regulon [11]. The differences in the bacterial cell wall and transcription profiles observed under certain environmental conditions in earlier studies were the basis for the work presented here as a manner to identify different bacillary populations with AR and IF.

Although acid-fast stains have been around for decades, the exact cellular component of *M. tuberculosis* recognized by the dyes is still being elucidated. Fuchsin, the main component of Ziehl-Neelsen and Kinyoun acid-fast stains, has been shown to stain the vastly complex lipid portion of the mycobacterial cell wall [31], [32], [33] and is also fluorescent [34]. However, less is known about the target of the combined auramine O-rhodamine B stain. Auramine O is believed to bind to mycolic acids [31] and nucleic acids [35], [36], [37]. Although rhodamine B has been used in numerous *M. tuberculosis* studies, the exact staining target in *M. tuberculosis* has yet to be discovered. The IF technique described in this study uses a rabbit polyclonal antibody raised against *M. tuberculosis* whole cell lysate to maximize detection sensitivity.

This study had a dual goal; to investigate whether various subpopulations of *M. tuberculosis* exist under different environmental conditions by staining for *M. tuberculosis* in culture, mice and guinea pigs; and to simultaneously detect multiple *M. tuberculosis* populations by detection of different targets with a combined IF-AR approach, and in addition, improving the sensitivity of the methods by using fluorescent signals. IF-AR staining revealed that there are at least three subpopulations of *M. tuberculosis* that exist within *in vitro* grown cultures and within mouse and guinea pig lung granulomas.

## Materials and Methods

### Ethics Statement

All experimental protocols were approved with written consent by the Animal Care Use Committee of Colorado State University (approval numbers ACUC #04-302A-06 and ACUC #06-221A-03) which abides by the USDA Animal Welfare Act and the Public Health Service Policy on Humane Care and Use of Laboratory Animals.

### Animals

Specific-pathogen-free, 8 to 10 weeks old female C57BL/6 mice and four to five month-old, female out-bred Hartley guinea pigs (approximately 500 g in weight) were purchased from the Charles River Laboratories (North Wilmington, Massachusetts). The C57BL/6-Ifngtm1ts gamma interferon gene-disrupted (GKO) mice were purchased from Jackson Laboratories (Bar Harbor, Maine). Animals were maintained in the biosafety level 3 biohazard facilities at Colorado State University, and were given sterile water, bedding, and enrichment for the duration of the experiments. The specific pathogen-free nature of the animal colonies was demonstrated by testing sentinel animals.

### Bacterial Isolates

The virulent *M. tuberculosis* strain Erdman (TMCC 107, purchased from ATCC), was grown as previously described [38]. Briefly, *M. tuberculosis* Erdman was grown to mid-log phase in Proskauer-Beck medium containing 0.01% Tween 80 (Sigma-Aldrich, St. Louis, Missouri) and stored in vials frozen at −70°C until use.

The H37Rv strain of *M. tuberculosis* (Trudeau Institute, Saranac Lake, New York) is used routinely in our laboratory for guinea pig infection studies [7], [39], [40]. *M. tuberculosis* H37Rv was grown from low passage seed lots in Proskauer-Beck liquid medium containing 0.05% Tween 80 to early mid-log phase and frozen in aliquots at −70°C until needed. Cultures were diluted in sterile water prior to use.

### *M. tuberculosis in vitro* oxygen depletion

The protocol used to grow *M. tuberculosis* under hypoxic conditions has been described [41] and is a slight modification of the method described by Wayne and Hayes [9] and Murugasu-Oei and Dick [42]. Briefly, mid-log-phase aerobic M. tuberculosis H37Rv cultures were diluted 100-fold in Dubos medium and transferred to tubes closed with sterile 8.0-mm silicone rubber septa (Aldrich, Milwaukee, Wisconsin). The cultures were grown at 37°C with slow stirring for 27 days. Control tubes contained methylene blue dye (1.5 µg/ml) which fades and finally disappears under anaerobic conditions, as described by Wayne and Hayes [9].

### Experimental infections

Mice were exposed to a low-dose aerosol infection (LDA) with *M. tuberculosis* strain Erdman (TMCC 107) in a Glass-Col inhalation exposure system (Glas-Col Inc., Terre Haute, Indiana) as previously described [38]. To verify lung bacterial uptake of 50 to 100 CFU per mouse, the bacterial load at one day post LDA was determined as indicated below. C57BL/6 mice were sacrificed 28 days and GKO mice were sacrificed 18 days post LDA by CO_2_ inhalation and spleens and left lung lobes were aseptically removed and bacterial loads determined as previously described [41]. Samples from the lower right lung lobe were also collected and processed for histological examination as indicated below.

Female Hartley guinea pigs were exposed to a low-dose aerosol of H37Rv strain of *M. tuberculosis* in a Madison aerosol chamber device known to result in approximately 20 lesions in the lungs as previously described [43], [44]. Guinea pigs were euthanized at 30 days post infection by sodium barbital injection (Sleepaway; Fort Dodge Laboratories, Indiana), and organs were aseptically removed and plated out as previously described [7]. Briefly, right cranial lung lobes were excised and homogenized in 4.5 ml of 0.85% sterile saline with a Kinematica Polytron tissue homogenizer (Brinkman Instruments Services, Westbury, New York). The bacterial load in each sample was determined and samples from each organ were also collected and processed for histological examination as indicated below.

### Bacterial load

The number of viable organisms in each organ sample was determined by plating serial dilutions of the organ homogenates on nutrient Middlebrook 7H11 agar plates (GIBCO BRL, Gaithersburg, Maryland). The plates were incubated at 37°C in ambient air for 4 weeks prior to the counting of the number of colony forming units (CFU) for each dilution.

### Histology

At the time of sacrifice, the lower right lung lobe was infused *in situ* with 10% neutral-buffered formalin and preserved for 48 hours until processed for histopathological assessment. At the time of processing, all tissues were embedded in paraffin, sectioned at 5 µm and stained with hematoxylin and eosin (H&E). The sections were examined by a veterinary pathologist and compared to the histopathology from previous animal studies completed in our laboratory [7], [41].

### Immunofluorescence

*M. tuberculosis* culture and lung tissue samples were examined by immunofluorescence as follows. Samples of *M. tuberculosis* culture were fixed in 4% paraformaldehyde (Electron Microscopy Supplies, Hatfield, Pennsylvania) in PBS for 30 minutes, washed in PBS then water and 1–2 µls were spotted on a glass slide. 5 µm sections of lung tissue were dewaxed in xylene and rehydrated through graded alcohols. The tissue sections and culture samples were then treated with 1 mg/ml lysozyme (Sigma-Aldrich, St. Louis, Missouri) and 30 U/ml achromopeptidase (Sigma-Aldrich, St. Louis, Missouri) in 10 mM Tris (pH 7.5) at 37°C for 40 min. Antigen retrieval was performed using the Retriever™ 2100 which pressure cooks at 121°C for 15 minutes using Target Retrieval Buffer solution S1699 (DAKO, Carpinteria, California). Blocking was performed with 1% goat serum in PBS (Biomeda, Foster City, California) for 30 minutes. The slides were incubated at 4°C overnight with rabbit polyclonal anti-TB whole cell lysate minus LAM (Antibody E293, CSU TB Vaccine Testing and Research Materials Contract, Colorado). Subsequently, the slides were washed using PBS and the antibody was detected with an Alexafluor 488 labeled goat anti-rabbit IgG (Invitrogen, Carlsbad, California). The slides were washed in PBS, mounted with ProLong® Gold antifade reagent (Invitrogen, Carlsbad, California) and photographed under a fluorescent microscope. When combining IF with auramine rhodamine, the coverslips were removed after IF and the slides were washed for 5 minutes in water and then stained with AR. The slides were again mounted and photographed under the same fluorescent microscope.

### Auramine-Rhodamine staining

*M. tuberculosis* culture and lung tissue samples were examined by auramine-rhodamine staining as follows. All samples were stained with TB Fluorescent Stain Kit T (Becton, Dickinson and Company, Sparks, Maryland) which is a combination of auramine O and rhodamine B. Samples of *M. tuberculosis* culture were fixed in 4% paraformaldehyde (Electron Microscopy Supplies, Hatfield, Pennsylvania) in PBS for 30 minutes, washed in PBS then water and 1–2 µls were spotted on a glass slide. Tissue sections were dewaxed in xylene and rehydrated through a graded alcohol series. Slides were then stained with a 1∶20 dilution of TB Auramine-Rhodamine T for 30 minutes. Slides were decolorized with TB Decolorizer TM (BD) until the stain was completely decolorized (3–5 minutes). Counterstaining was performed with hematoxylin QS (Vector Laboratories, Inc., Burlingame, California) for approximately 5 seconds for tissue sections or TB potassium permanganate (BD) for *in vitro* samples. The slides were washed with ddH_2_O and mounted using ProLong® Gold antifade reagent (Invitrogen, Carlsbad, California) and reexamined using the same microscope and camera as used for IF stained slides.

### Optimization of combined IF and AR

Extensive optimization was completed before applying IF-AR on tissue samples. Different protocols were evaluated where the sequence of AR and IF on the slides was tested either alone or in combination. Preliminary results showed that the intensity of the IF was severely reduced when the AR staining preceded the IF. In addition, AR also interfered with IF by reducing the signal when AR was applied last. Therefore, the final protocol was as follows; IF was performed first and the slides were photographed, then the coverslips were removed the slides were stained by AR and photographed again. To test this protocol and ensure that IF staining did not interfere with the subsequent AR staining, serial sections of a primary granuloma were cut and numbered. Odd numbered sections were stained with IF-AR and even numbered sections were stained with AR alone. The results showed that after IF and AR combined staining no loss of AR signal was observed when compared to AR alone.

### Fluorescent *in situ* hybridization

FISH was performed using three antisense ssDNA probes targeting *M. tuberculosis* 16s ribosomal RNA. Probes used were *M. tuberculosis* 187 (MTB187) [21], *M. tuberculosis* 223 (MTB223) (5′-CCCACACCGCTAAAGCGC-3′) and *M. tuberculosis* 1284 (MTB1284) (5′-GAGACCGGCTTTTAAGGATTCG-3′). Probes MTB223 and MTB1284 were newly designed in our laboratory using ARB software [45]. The positive and negative control probes, EUB338 and non-EUB338, have been previously described [46], [47]. All the sense and antisense probes were purchased pre-labeled with Alexafluor 568 nm from Invitrogen (Carlsbad, California) and were used at final concentration of 1 µg/ml.

*M. tuberculosis* culture and lung tissue samples were examined by FISH as follows. Firstly the tissue sections were dewaxed and rehydrated through graded alcohols and finally incubated in PBS. At this stage, all culture and tissue sections received the same treatment. Samples were permeabilized for 1 hour at 37°C using 10 mg/ml lysozyme (Sigma-Aldrich, St. Louis, Missouri) in PBS. Subsequently, the tissue sections were incubated with the prehybridization buffer for 1 hour at 37°C. Prehybridization buffer contained 20% 20X SSC (Boston Bioproducts, Worcester, Massachusetts), 20% dextran sulphate, 50% formamide, 1% 50X Denhardts solution (Sigma-Aldrich, St. Louis, Missouri), 2.5% 10 mg/ml Poly A, 2.5% 10 mg/ml ssDNA, 2.5% 10 mg/ml tRNA (Invitrogen, Carlsbad, California). After a quick rinse in 2X SSC, hybridization buffer (prehybridization buffer with probe) containing the three fluorescent rRNA probes were added to the sections, heated briefly for 5 minutes at 75°C and then incubated overnight at 37°C. All prehybridization and hybridization incubations were performed in a Hybaid OmniSlide *in situ* Thermal Cycler System (Thermo Fisher Scientific, Waltham, Massachusetts). Thereafter, the sections were washed using a Hybaid Heated Wash Module (Thermo Fisher Scientific, Waltham, Massachusetts) according to the following protocol; first a quick wash in 1X SSC at RT to remove the coverslips, followed by two washes for 15 min at 55°C with 1X SSC and another two washes for 15 min at 55°C in 0.5% SSC and finally ending with one wash for 10 min at RT in 0.5X SSC. The sections were counterstained with DAPI (Sigma-Aldrich, St. Louis, Missouri) and mounted in Prolong Gold (Invitrogen, Carlsbad, California).

### Fluorescent Microscopy

Photographs were taken on a Nikon Eclipe 80i with DAPI, FITC and TRITC filter cubes and an Optronics Microfire color fluorescent camera. Multiple photographs were taken under different focal planes when necessary and combined using Nikon NIS Elements AR 3.0 software. For confocal microscopy, a Zeiss Axio Observer confocal microscope was connected to a LSM510 META confocal laser source. A 488 nm argon laser was used with emission filter 505–550 nm. Images were captured and analyzed using Ziess LSM image analyzer version 4.0.

## Results

### Detection of *M. tuberculosis* in culture

**Figure 1:**
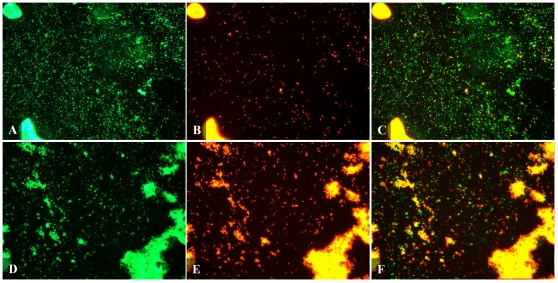
Fluorescent micrograph of Mtb detected by IF and AR in culture. Mtb was detected by IF (A,D), AR (B,E) and combined IF-AR (C,F) in culture grown to stationary phase (A–C) and culture grown under hypoxic conditions (D–F). Culture was smeared on a poly-L-lysine coated slide and fixed with paraformaldehyde. Rabbit polyclonal antibody against whole Mtb cell lysate was applied for 1 hour at room temperature and detected using an Alexafluor 488 labeled secondary antibody (green). Slides were then stained with AR (orange/red) for 30 mins then destained. Pictures were taken using FITC and TRITC filters at 400× magnification. Bacilli seen here are mostly single cells but clumps of varying sizes are common.

Samples were obtained from cultures of *M. tuberculosis* grown aerobically to stationary phase for 55 days and cultures grown under hypoxic conditions (which is hypothesized to mimic hypoxic conditions in necrotic granulomas) for 27 days as described by Wayne [9], [41]. These samples were stained by IF. In stationary phase culture, the IF+ bacteria yielded strong, intense, positive fluorescence (Figure 1, A). Interestingly, the IF signals varied between bacilli. Signals ranged from full rod shapes to a punctuated form with one to several intense dots or focal points. In cultures grown under hypoxic conditions the punctuated signals were more pronounced (Figure 1, D). This punctuate staining was also seen in all *in vivo* samples.

Cultures were then stained with AR which showed highly fluorescent orange-red bacteria. This staining was uniform throughout the bacillus and also defined its rod-shaped form (Figure 1, B&E). The bacilli predominantly existed as single organisms however various sized aggregates were frequently seen. The latter is a common characteristic of culture-grown *M. tuberculosis*.

We then evaluated whether multiple phenotypic populations could be observed under various culture conditions when combining IF with AR. This was done by comparing stained samples obtained from aerobically grown stationary culture and culture grown under hypoxic conditions. Samples were stained with the combined IF-AR procedure to simultaneously detect protein and acid-fast targets. Slides were first stained by IF and fluorescence signals (green color) were captured using a FITC filter. The slides were subsequently stained with AR (red color) and then a TRITC filter was used to capture the AR fluorescence signals. The two photographs were combined and analyzed using NIS Elements software (Nikon). Yellow signals occur when bacilli are detected by both IF and AR.

When IF-AR was applied to both types of culture samples, more bacilli were detected by IF than AR in both stationary and hypoxic culture. In samples from stationary phase culture, 100% of bacilli that stained positive by AR (AR+) were also positive by IF (IF+) giving a yellow color (Figure 1, C). Therefore two populations of *M. tuberculosis* were found in stationary culture; bacilli that are detected by IF alone (green color) and bacilli that are detected by IF-AR (yellow). When bacterial samples grown under hypoxic culture conditions were stained by IF-AR (Figure 1, F), there was a noticeable difference when compared to samples from stationary phase culture. IF again detected more bacilli than AR but the number of AR positive bacilli was higher in hypoxic culture than in stationary culture. Interestingly, about 30% of AR stained bacteria in hypoxic culture were positive exclusively by AR (red color) and were not detected by IF. This result showed three populations of *M. tuberculosis* in hypoxic culture; bacilli that were detected by IF alone (green and IF+ AR-), AR alone (red and IF- AR+) or by the combined IF-AR technique (yellow and IF+ AR+). This dual IF-AR technique detected a higher total number of stained bacilli in hypoxic culture than using either technique alone.

**Figure 2:**
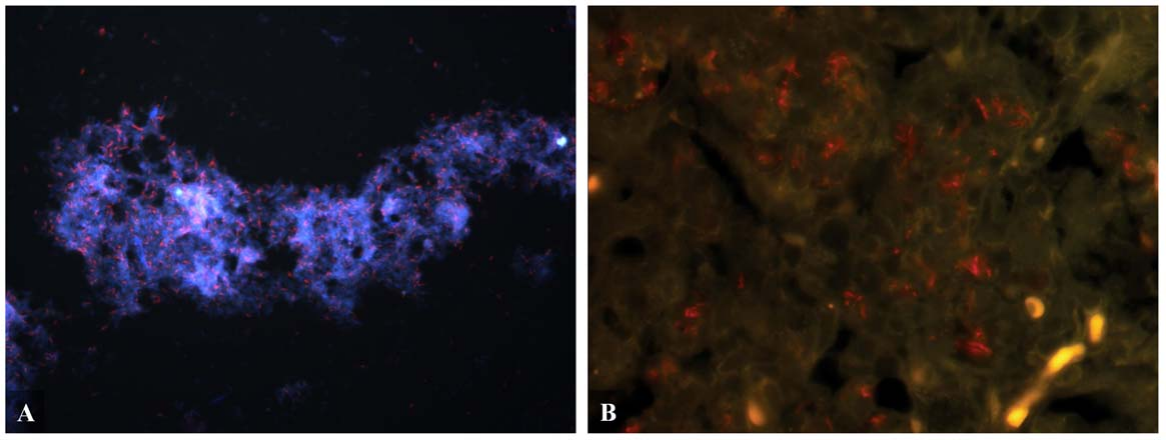
FISH detecting Mtb in culture and mouse lung tissue. Mtb (red) was detected in (A) hypoxic culture and (B) GKO mouse lung tissue at 4 weeks post infection. Mice were exposed to a low-dose aerosol infection (LDA) with *M. tuberculosis* strain Erdman (TMCC 107). 5 µm formalin fixed paraffin embedded sections were dewaxed and rehydrated. Three fluorescent Alexafluor 568 nm labeled ssDNA probes designed to detect the 16 s rRNA subunit were hybridized overnight at 37°C after digesting sections for 1 hr with lysozyme at 10 µg/ml. Sections were counterstained with DAPI and mounted. Pictures were taken using FITC and TRITC filters at (A) 400× magnification and (B) 1000× magnification.

Finally, bacterial samples from stationary and hypoxic cultures were also studied using FISH to detect the 16 s subunit of *M. tuberculosis* rRNA. Evaluation of the specificity of the two newly designed *M. tuberculosis* probes (MTB223 and MTB1284) was performed by fixing *M. tuberculosis* culture onto glass slides and detecting with FISH. When used to detect rRNA with all three probes (MTB187, MTB223 and MTB1284) the bacteria appeared as positive rod-shaped signals (Figure 2, A) similar to AR stained bacilli although the signal was not as intense.

### Detection of *M. tuberculosis* in murine models of infection

Using the different detection methods, we investigated the presence of multiple phenotypes of *M. tuberculosis in vivo* in different locations in the lung. To demonstrate this, two mouse strains were used which are currently used in our laboratories for drug testing [50]. Immunocompetent C57BL/6 mice and the immunocompromised GKO mice were infected with *M. tuberculosis* by LDA as previously described [38].

Lung tissue sections obtained from C57BL/6 and GKO infected mice at the time of sacrifice were first stained using H&E. The histopathology of C57BL/6 and GKO mice has been previously described [48], [51]. In brief, at 28 days post LDA the granulomatous lung tissue of C57BL/6 mice consists of large lymphocyte aggregates surrounding multiple, smaller accumulations of epithelioid macrophages. In GKO mice at 18 days post LDA there are coalescing foci of multiple lesions that form considerable areas of inflammation. These granulomas differ from guinea pig granulomas as they do not form an organized hypoxic necrotic core [52], [53], [54].

**Figure 3:**
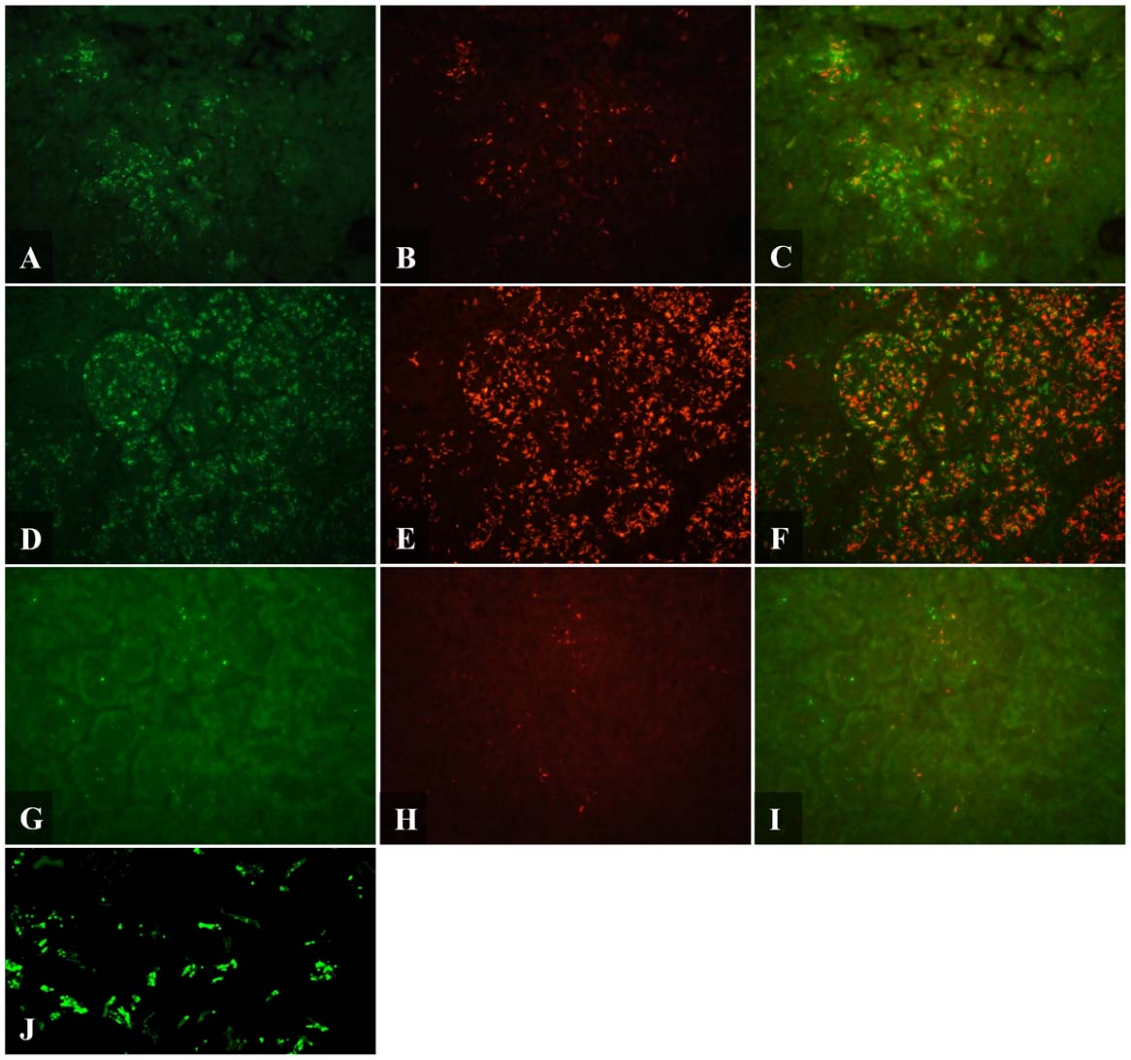
Fluorescent micrograph of Mtb detected by IF and AR in mouse and guinea pig. Mtb was detected by IF (A,D,G,J), AR (B,E,H) and combined IF-AR (C,F,I) in C57BL/6 mouse lung (A–C), GKO mouse lung (D–F) and guinea pig lung (G–I). 5 µm formalin fixed paraffin embedded sections were dewaxed, rehydrated and detected by IF. Rabbit polyclonal antibody against whole Mtb cell lysate was applied overnight at 4°C and detected using an Alexafluor 488 labeled secondary antibody (green). Slides were then stained with AR (orange/red) for 30 mins then destained. Pictures were taken using a FITC and TRITC filter at 400× magnification. (J) Confocal micrograph of Mtb detected by IF in GKO lung tissue at 1000× magnification with 1.6× digital zoom.

When lung tissue sections from both C57BL/6 and GKO mice were stained by IF, the bacilli most frequently stained in a punctuate manner (Figure 3, A&D) similar to that seen in IF stained hypoxic culture (Figure 1, D). Uniform, rod-shaped staining was an infrequent event unlike auramine-rhodamine staining which consistently produced complete rod-shaped bacilli (Figure 3, B&E). In both mouse models, bacilli were associated with granulomatous tissue and IF positive bacilli were in the same location as AR positive bacilli. IF detection revealed that GKO mice (Figure 3, D) had larger numbers of positive bacteria than C57BL/6 mice (Figure 3, A).

To further examine the punctuate features of this staining we analyzed tissue sections using confocal microscopy which revealed varied signals ranging from a weak bacillus outline with single or several intense dots to full rod shaped bacilli (Figure 3, J). This punctuate characteristic of the IF staining demonstrated that the antibody binding was more concentrated in some areas than others. Confocal micrographs also demonstrated that the antibody was penetrating the tissue section on the slides as bacilli were stained equally throughout the depth of the tissue.

Similar sections from mice were then stained by AR. Bacilli were mostly found intracellular and strongly associated with granulomatous tissue (Figure 3, B). Lung sections of GKO mice stained by AR also revealed that bacilli were associated with inflammatory lesions which contained exceptionally large numbers of intracellular and extracellular AR positive bacilli (Figure 3, E). In general, bacilli stained by AR exhibited a uniform, strong staining pattern however quite a small fraction were very faint.

The combined IF-AR procedure was then applied to murine lung tissue. Similar to that seen in hypoxic culture, IF-AR resulted in the detection of three populations of *M. tuberculosis* in both C57BL/6 (Figure 3, C) and GKO mice (Figure 3, F). Bacilli were either detected by IF alone (green), AR alone (red) or concurrently by both techniques (yellow). The three populations were found homogenously distributed throughout the lung granulomas. These results indicate that within one environment, *M. tuberculosis* can exhibit different phenotypes. We intended to determine the total number of bacteria per field, however due to the close proximity and high numbers of bacilli, accurate enumeration of the bacteria was not possible. Also it was not possible to capture all the signals in one plane of focus. Therefore photographs were taken at varying focal planes which were overlaid and analyzed using NIS Elements software. This analysis revealed that when using either IF or AR alone, the number of bacilli was approximately 40–60% lower to that observed in a tissue section stained by IF-AR.

When AR or IF were applied individually, a varied signal intensity between bacilli was observed ranging from quite strong to quite weak. Most bacilli were easily seen whereas others were close to the minimum level of detection suggesting that by a single technique not all populations were being detected. If some bacilli were quite weakly stained then it could be possible that others were below the limits of detection hence the need to combine the two complementary detection techniques.

FISH was used to detect *M. tuberculosis* in infected mouse samples using fluorescent *M. tuberculosis* probes MTB187, MTB223 and MTB1284. Positive rod-shaped signals were observed in GKO mice (Figure 2, B). The FISH signals observed *in vivo* were not as intense as the results obtained *in vitro* (Figure 2, A). The FISH signals in GKO mice (Figure 2, B) were also noticeably weaker than the signals obtained by AR and IF (Figure 3, F).

### Detection of *M. tuberculosis* in the guinea pig model of infection

The guinea pig model was studied because of the presence of hypoxic, necrotic granulomas after *M. tuberculosis* infection, which are similar to those seen in human pulmonary infection [5], [48], [49].

When detected by IF, bacilli were primarily located extracellularly within the necrotic core of primary granulomas and with lesser numbers in the surrounding fibrotic rim. Very few IF+ bacilli were found in non-necrotic granulomas. Bacilli predominantly existed as single organisms with small microclusters of approximately 2–10 bacilli (Figure 3, G). As seen in cultures and in mice, bacilli in the guinea pig mainly stained by IF in a punctuate manner with some complete rods. The range of staining intensity was similar to that seen in mouse tissue with some bacilli easily visible whilst others were quite weak with a single focal point of fluorescent signal.

In the current study, at 30 days post infection, bacilli in primary lesions are predominantly extracellular, located within the necrotic core of granulomas and within the surrounding fibrotic rim. Similar to IF staining, bacilli largely existed in the guinea pig as single organisms but also as small microclusters containing 2–10 bacilli (Figure 3, H). In the secondary lesions, there were very low numbers of AR positive bacteria which were found intracellular. As found in mouse tissue, bacilli stained intensely by AR however there was a very small proportion that stained quite weakly.

Combining IF and AR revealed fewer bacilli numbers in the guinea pig lung sections than that seen in mouse tissue, which is as expected due to lower bacterial numbers per section as determined by colony forming unit counts. The three different *M. tuberculosis* populations seen in mice were also detected in guinea pig tissue; bacilli that stain with either AR or IF alone and bacilli that stain simultaneously by both techniques (Figure 3, I). Similar to the result found in mice, the combined IF-AR technique detects a higher total number of bacilli compared to using either IF or AR alone. These multiple populations were mostly found homogeneously distributed within the necrotic core of granulomas with a smaller proportion in the surrounding acellular rim.

When FISH was applied using three fluorescent *M. tuberculosis* probes, *M. tuberculosis* could not be visualized in guinea pig tissue sections that were shown to harbor bacilli by IF and AR. This result contrasts with the strong FISH results seen in cultures and moderate signals in mice.

## Discussion

The aim of this work was to investigate whether the microenvironment plays a role in determining the phenotype of *M. tuberculosis*. To achieve this objective, current detection techniques were first assessed for their ability to detect *M. tuberculosis* in culture and tissue samples. The detection techniques used for identification were IF which is directed towards bacterial surface proteins and AR which stains cell wall components and FISH which detects rRNA. We introduced a new approach to studying *M. tuberculosis* phenotypes by using IF-AR simultaneously. By combining both methods we not only intended to detect both acid-fast and non acid-fast bacteria, we also aimed at elucidating *in vivo* the ratios of both subpopulations in the different locations of the lung lesion.

The findings in this study demonstrate that in all samples *M. tuberculosis* stains in a punctuated manner by IF and in a uniform manner using the acid-fast AR method. The uniform, intense positive stain obtained by AR enabled slides to be assessed easier and quicker when compared to slides stained by IF. Also, there was slight background tissue autofluorescence associated with IF whereas AR produces none. The autofluorescence was likely caused by overlaying multiple (2–3) IF photographs of varying focal planes.

Bacilli were shown to stain either exclusively by IF (green), exclusively by AR (red) or by both techniques concurrently (yellow). In samples from stationary phase culture, two populations of green and yellow bacilli were observed. The green bacilli were IF positive, acid fast negative (IF+ AR−) whilst the yellow population was IF+ AR+. However in hypoxic culture, three different phenotypic populations of green, red and yellow were revealed indicating that hypoxia induces phenotypic changes leading to a diverse population being detected exclusively by acid-fast AR (and therefore IF− AR+). Although previous studies have presented strong evidence indicating phenotypic alterations, this is the first study documenting the presence of multiple *M. tuberculosis* populations within one microenvironment.

In mice and guinea pigs, all three subpopulations of bacilli were found homogeneous over the lung lesions studied. We estimate that, on average, about an equal proportion were present of the green and red individually stained bacilli. In addition to the single stained bacilli another subpopulation of dual stained yellow bacilli was observed which was a smaller population than either green or red. It has been a general thought that *M. tuberculosis* can alter its metabolism depending on its environment in order to survive. We hypothesized that this could be reflected in seeing different subpopulations depending on the various microenvironments mimicked *in vitro* and *in vivo* conditions. However, the results showed that under several *in vitro* conditions *M. tuberculosis* is always present as two or three different subpopulations. The same was observed *in vivo*, where even in the cores of the hypoxic, necrotic lesions not only one single population but three different populations of *M. tuberculosis* were visualized by the dual staining technique. The results suggest that *M. tuberculosis* may always be present in various stochastic phenotypes which could be seen as an ultimate survival strategy to adapt quickly to altering conditions [3]. Phenotypic heterogeneity has been linked to persistence of microbes under varying microenvironments [55], [56] and in the case of *M. tuberculosis* persistence may be partly responsible for the difficulty of treating tuberculosis disease [16].

It is unclear at this time what these differences in cell wall compositions are as the exact targets of AR and IF have not been fully determined. However, in our experience, rhodamine B alone stains *M. tuberculosis* quite readily in culture and tissue sections and also stains purified cell wall components (unpublished data). Indeed, a thorough evaluation of acid-fast staining is necessary to identify the staining targets and therefore define these three different populations. Current studies are underway in our laboratory to determine which cell wall component(s) are stained by fuchsin, auramine and rhodamine.

One population of bacilli was acid-fast negative. A decrease in acid-fast staining is reportedly related to mycolic acid composition. Various reports have shown that *M. tuberculosis* can alter its cell wall composition and this can lead to a loss of acid-fastness [19], [20], [24], [25], [26]. In earlier papers it has been suggested that an intact or fully encompassing cell wall is required for retention of acid-fast dyes. However we have digested the cell wall sufficiently to the allow horseradish peroxidase enzyme (40 kDa) to penetrate and yet the bacilli were still strongly acid-fast positive (unpublished data).

Another population in this study was IF negative. The reason why a population is IF negative is unclear. One reason is that the anti-whole cell lysate antibodies used in this study were raised against bacilli that were in late log stage growth. It is possible that under hypoxic conditions, *M. tuberculosis* expresses a different set of surface proteins or reduces surface protein expression as the bacilli enter what is likely to be a dormant state. We are currently generating polyclonal antibodies against different stages of bacilli in order to address this question. Another reason may be protection by the polysaccharide rich capsule of *M. tuberculosis* which has been described by others [57]. This capsule may mask the bacterial surface proteins from the antibody. An alternative explanation is that these IF negative bacilli are in fact dead and the surface proteins were degraded more quickly than the mycolic acid cell wall leaving an acid-fast ‘shell’. As for the IF positive bacterial population, the exact proteins detected are not known. The rabbit polyclonal anti-whole cell lysate antibodies used by IF in this study have numerous protein targets as determined by western blot (data not shown) but it is not known which proteins were detected in this study and positive detection by western blot does not always reflect immunodetection in tissue. Although we report the presence of multiple phenotypes, we cannot fully explain the biological relevance as the staining targets have not been completely identified.

In the current study FISH was assessed for its ability to detect all populations of *M. tuberculosis in vitro* and *in vivo* by targeting *M. tuberculosis* 16 s ribosomal RNA. FISH yielded strong signals in hypoxic culture but resulted in only moderate signals in mice and failed to detect *M. tuberculosis* in guinea pigs. Of importance, these results indicate that the bacilli under the various *in vitro* and *in vivo* conditions are different in their ability to be identified by FISH. One can speculate as to why there was no signal in guinea pig tissues whereas a moderate signal was observed in mice. The lack of FISH signals in necrotic granulomas of guinea pigs could be due to a reduction of *M. tuberculosis* rRNA numbers in the bacteria within the necrotic lesions to below the limits of FISH detection. An alternative explanation is that the cell wall of the bacteria in these hypoxic regions might have changed and thickened, and therefore have become far less permeable to the probes used in FISH. It could also be a combination of both explanations. Of significance, these results show that the bacilli in mice and guinea pigs are not identical. Currently, we are trying to study whether the bacilli in the primary granuloma show a thickened or altered cell wall using electron microscopy, a change that has been reported in *M. tuberculosis in vitro* [26]. In the guinea pig model, most bacilli were observed within the necrotic regions of the primary granulomas by IF and AR staining. The necrotic granuloma has been shown to be hypoxic [7], [8], however the FISH signals were quite strong in samples from hypoxic *M. tuberculosis* culture indicating that hypoxia alone does not decreased rRNA FISH signals. This result was also seen in another *in vitro* study which showed that *M. tuberculosis* rRNA levels were stable throughout log phase, microaerophilic nonreplicating phase I (NRP-1) and anaerobic NRP-2 stages [58]. Our results show that *in vitro* grown bacilli under hypoxia are not identical to those found in the hypoxic, necrotic lesions of the guinea pig model.

In the present study we mainly concentrated on the cross species comparison of the location of different subpopulations of *M. tuberculosis in vivo* at a single time point after infection. It would be interesting to study whether the ratios of these different phenotypes would change over the progression of disease. An earlier published study by Seiler *et al* indicates that this could be the case [19]. In mice, while bacilli numbers remained constant over time by culture and IHC, the numbers of acid-fast (carbolfuchsin and AR) organisms were drastically reduced. Current studies are expanding this work to enumerate bacteria in drug treated and untreated guinea pigs over time which includes early and late stages of infection.

The discovery of multiple phenotypes of *M. tuberculosis* within the same microenvironment *in vitro* as well as *in vivo* reveals a new challenge. Several studies are underway trying to elucidate the microenvironments surrounding the bacilli and investigating the metabolic changes of the bacilli in these microenvironments by genomic, proteomic, metabolomic and lipidomic methodologies. A vast array of information will be gained by these studies. Although the field has started to recognize that these studies should not look at entire lungs because of differences between lesion types in most larger animal models, the results presented here indicate that the task might even be far more complicated. In order to assess an accurate measurement of protein, lipid or genetic profile of the bacilli it might be necessary to look at a per-cell basis with new technologies.
